# Structure-Activity Relationship for Fe(III)-Salen-Like Complexes as Potent Anticancer Agents

**DOI:** 10.1155/2014/745649

**Published:** 2014-04-06

**Authors:** Zahra Ghanbari, Mohammad R. Housaindokht, Mohammad Izadyar, Mohammad R. Bozorgmehr, Hossein Eshtiagh-Hosseini, Ahmad R. Bahrami, Maryam M. Matin, Maliheh Javan Khoshkholgh

**Affiliations:** ^1^Department of Chemistry, Faculty of Science, Ferdowsi University of Mashhad, Mashhad 9177948974, Iran; ^2^Institute of Biotechnology, Ferdowsi University of Mashhad, Mashhad 9177948974, Iran; ^3^Department of Chemistry, Mashhad Branch, Islamic Azad University, Mashhad 9187147578, Iran

## Abstract

Quantitative structure activity relationship (QSAR) for the anticancer activity of Fe(III)-salen and salen-like complexes was studied. The methods of density function theory (B3LYP/LANL2DZ) were used to optimize the structures. A pool of descriptors was calculated: 1497 theoretical descriptors and quantum-chemical parameters, shielding NMR, and electronic descriptors. The study of structure and activity relationship was performed with multiple linear regression (MLR) and artificial neural network (ANN). In nonlinear method, the adaptive neuro-fuzzy inference system (ANFIS) was applied in order to choose the most effective descriptors. The ANN-ANFIS model with high statistical significance (*R* ^2^_train_ = 0.99, RMSE = 0.138, and *Q* ^2^_LOO_ = 0.82) has better capability to predict the anticancer activity of the new compounds series of this family. Based on this study, anticancer activity of this compound is mainly dependent on the geometrical parameters, position, and the nature of the substituent of salen ligand.

## 1. Introduction

Despite several efforts in the treatment of cancer, because of several limitations that using medications has, this disease became a big problem for the health of societies. The purpose is to develop medications with more anticancer activity and less toxicity than the present medications. Metallic compounds have been widely studied due to their major role in biological activities. Since the introduction of cisplatin as anticancer medication, a comprehensive study has been performed on the metal complexes and the medicinal features of these compounds [1–3]. Currently, metal complexes of different transition metal are preferred candidates for the treatment of different sort of cancer. Medicinal inorganic chemistry can employ different strategies in the development of unique properties of metal ions for design of new anticancer drugs. However design, synthesis, and structural characterization of metallodrugs have attracted a lot of interest due to their applications in anticancer fields. The performance of these compounds is explained on the basis of many mechanisms including intercalation, inhibition of DNA and RNA [4–8]. Lipinski, Murcke, and coworkers had an important role in concentrating on the importance of features of such medication on the basis of its shape. The anticancer activity of the metallic complexes has also an adjacent relationship with the type of central metal and binding ligand to it [1, 9]. Meanwhile, bis(salicylidene)ethylenediamine ligands are subject of the study for a long time and several of them including various metals have been synthesized [9–26]. Iron-salen complexes have been studied since 1931 because of the physical and biological features and it has been cleared that salen complexes of F(III) have anticancer features on the MCF7 cells [27]. Metallosalens damage DNA/RNA in vitro. Iron-salen derivatives produce hydroxyl radicals in the presence of reducing agent of dithiothreitol (DTT) and damage DNA [28, 29]. The probability of discovering a natural medication which did not need correction or probability of providing a defined compound as a medication is too rare. Today, the molecular and chemical computing models are used in designing new medications which resulted in saving time and cost and designing medications with more potential. Among various computational methods, QSAR has a remarkable role in designing a medication. In fact, underlying basis of SAR (structure-activity relationships) focuses on the elucidation of structure and biological effects but QSAR attempts to form a quantitative relationship between them [30–33]. QSAR models are mathematical equations which relate the chemical structure of compounds to their biological activity [34–37]. There are theoretical pathways which are used to encode the information of the molecular structure into numbers to acquire these equations [38, 39]. The relation of construction activity of anticancer Fe(III)-salen-like complexes had been studied a lot in the past. One important viewpoint of the researchers in the past works showed that the nature of substitutes and bridge between D-amino groups play the main role in defining the anticancer feature of these compounds. This happens in a way that increases in the aromaticity of D-amino bridges and replacing of hydroxyl groups with methoxy (except some items) will lead to increase in anticancer feature [2, 28]. But, considering available information resources, there is no report, in quantities viewpoint, QSAR, to show this relation more exactly and with more details.

In this paper, what we considered has been searching for QSAR relation for Fe(III)-salen and salen-like complexes with linear and nonlinear methods and designing a model with high statistical significance to predict anticancer activity of new compounds series of this family. QSAR analysis in this study is based on the mathematical relationship between biological activity and structural geometric, quantum-chemical, electronic, and spectral features. For this purpose, substitutes on salen ligand and chloride ligand replacement with N-heterocyclic ligands were studied. With this replacement, anticancer activity will be changed in a wide range and modeling is a tool used to understand and predict diverse activities. Two main goals were pursued in this work: (1) the study of the influence of the increase in aromatic rings on the bridge between D-amino groups on the anticancer activity and (2) the study of the influence of the change in the position and nature of the salen ligand on the anticancer activity. The resulting equations adequately describe the biological activity of these complexes and assign important descriptors of compounds for efficient anticancer activity.

## 2. Materials and Methods

### 2.1. Data Set

**Figure 1:**
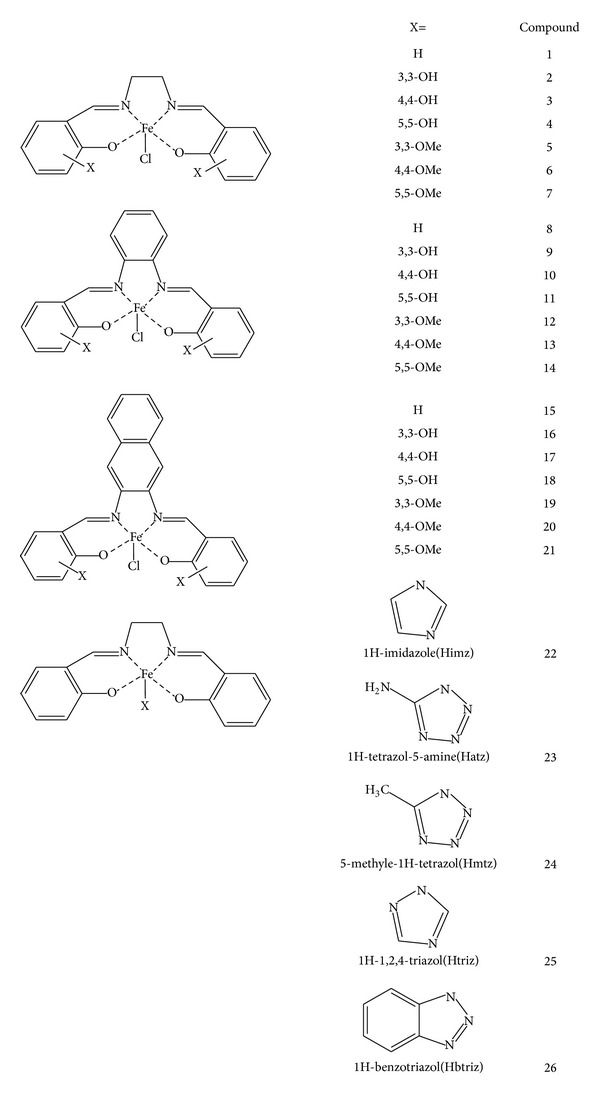
Structure of the complexes studied.

Biological data which were used in this work were the anticancer activity of 26, Fe(III)-salen and salen-like complexes against human breast cancer cell line (MCF7) in terms of IC_50_. The quantities of IC_50_, under the same conditions, were collected from previous studies [2, 27, 28]. The structures of studied compounds and quantities of their anticancer activity have been reported in Figure 1 and Table 1, respectively. The activity data have been converted into logarithm units (PIC_50_) then were used for modeling.

### 2.2. Geometry Optimization and Molecular Descriptors Calculation

The optimized 3D geometry of the molecules was achieved using Gaussian 03 software as well as B3LYP technique and LANL2DZ basis set. This method presents satisfactory results for the optimization of the 3D geometry of the metal complexes [40, 41]. Dragon packages, Gaussian 03, and AIM were used for calculation of molecular descriptors [41, 42]. A pool of descriptors was calculated by Dragon software for each molecule including parameters of all types such as constitutional, topological, geometrical, GETAWAY, WHIM, 3D-MoRSE, Molecular Walk Counts, BCUT descriptors, 2D autocorrelations, aromaticity indices, randic molecular profiles, radial distribution functions, functional groups, atom-centered fragments, empirical and properties [42]. In addition, highest occupied molecular orbital (HOMO), lowest unoccupied molecular orbital (LUMO), dipole moment, natural charge, shielding NMR, and total energy were calculated by DFT method. Chemical hardness (*η*), chemical softness (*σ*), chemical potential (*μ*), and electrophilicity (*ω*) were calculated according to the equations [38, 43]. Charge density (*ρ*(*r*)) and Laplacian of the electronic charge density ∇^2^ *ρ*(*r*) which were calculated using AIM software were based on the quantum theory of atoms in molecules [44].

### 2.3. Descriptor Selection

Computed descriptors and empirical data were analyzed using SPSS software [45]. These data were put in a quadratic matrix in which its order is equal to the number of molecules and descriptors. Among descriptors, those which had more correlation with anticancer activity were saved and the others were omitted. In recent years finding the most efficient descriptors from a pool of variables plays a fundamental role in QSAR studies [46, 47]. In this work, finding the most efficient descriptors was performed with linear and nonlinear methods; finally, created models were compared. Selection of the most efficient attributes and obtaining of the final equation are very convenient by linear techniques. In this work, QSAR equations between independent descriptors and PIC_50_ empirical parameters were obtained as a response in a way that each category of descriptors was considered individually and the most efficient descriptors by multiple linear regression- (MLR-) stepwise were selected. Since in the nonlinear method ANN cannot select the most significant descriptors, ANFIS algorithm, a developed algorithm based on neural network and fuzzy logic, was used. This algorithm can characterize extremely nonlinear functions. ANFIS was used here for investigation; the most effective parameters in a target function and the most effective descriptors were selected [48].

### 2.4. Model Development

In this work MLR was employed as linear technique and ANN as nonlinear ones for the QSAR models.

#### 2.4.1. MLR-Stepwise

In this stage study of structure and activity relationship was performed with multiple linear regression (MLR) in SPSS software [49, 50]. The most efficient descriptors by multiple linear regression- (MLR-) stepwise were selected. Then five descriptors were selected by this procedure [51, 52].  Table 2 represents the selected variables and their chemical meanings. The correlation matrix among these descriptors is shown in Table 3. Find correlation between PIC_50_ and five descriptors is given by

(1) PIC50=−8.991+1.354CIC1+11.336H8m −2.045MATS8e+22.943G3s−2.032Mor28u.

#### 2.4.2. ANN-ANFIS

**Figure 2:**
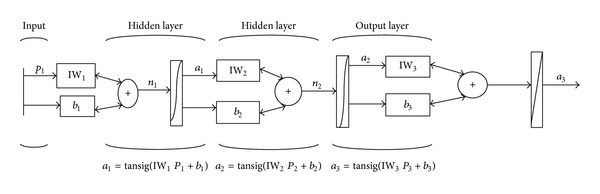
The architecture of feedforward neural network.

Artificial neural networks are generally used for nonlinear regressions [53, 54]. The most effective descriptors were selected by using ANFIS algorithm. For comparing linear and nonlinear selection in Table 4, the most efficient descriptors from the pool of descriptors which are selected by ANFIS models and their chemical meanings have been represented. In this study multilayer feed-forward (MLFF) network with back-propagation (BP) learning was employed and its overview is shown in Figure 2. We use the Matlab 7.0 program in these calculations [48, 55]. For training of neural network, obtained descriptor and anticancer activity were used as inputs and outputs, respectively. After the training of the network, the resulting ANN model was used to predict the activity of the test set compounds. Normalized inputs and outputs have better effect on training. The network includes some hidden layers with sigmoid neurons and final linear layer. The function of nonlinear transmit to the network provides the ability of learning linear and nonlinear relationship between inputs and outputs and the external linear layer enables the outputs to be out of the range of −1 and +1. With performing the network, the statistical weights of each of the descriptors will change alternatively till the error between anticipated values of PIC_50_ and the values of empirical PIC_50_ (target vector) is minimized. Several models with various numbers of hidden layers and neurons were designed and they are optimized by a systematic search method. The best network model with 3 layers and 9, 8 and 1 neuron was selected. The network was trained with Levenberg-Marquardt (LM) algorithm [56, 57].

#### 2.4.3. Validation of QSAR Models

Validation process is a necessary step in QSAR. In fact the QSAR models were validated by the calculation of the statistical terms (correlation coefficient *R* ^2^, cross-validation *Q* ^2^, standard error of prediction *S*, root mean square error RMSE, etc.). Cross-validation is the statistical method of partitioning a sample of data into training set and test set. The test set was used for external validation. One of the cross-validation methods is LOO where one object at a time is removed from the data set and then predicted by generated model. The *Q* ^2^_LOO_ was calculated using

(2) $$\begin{array}{c} Q^{2}=\frac{1-\text{Press}}{\text{SSY}}, \end{array}$$

where Press = ∑(*Y*_pred_−*Y*_actual_)^2^ and SSY = ∑(*Y*_actual_−  *Y*_mean_)^2^ and where *Y*_pred_ is a predicted value of activity, *Y*_actual_ is an actual or experimental value of activity, and *Y*_mean_ is the mean activity value [38]. We apply cross-validation method to determine that QSAR models have ability to correctly predict the biological activities of new compounds. Results have been reported in Table 5.

## 3. Results and Discussion

**Figure 3:**
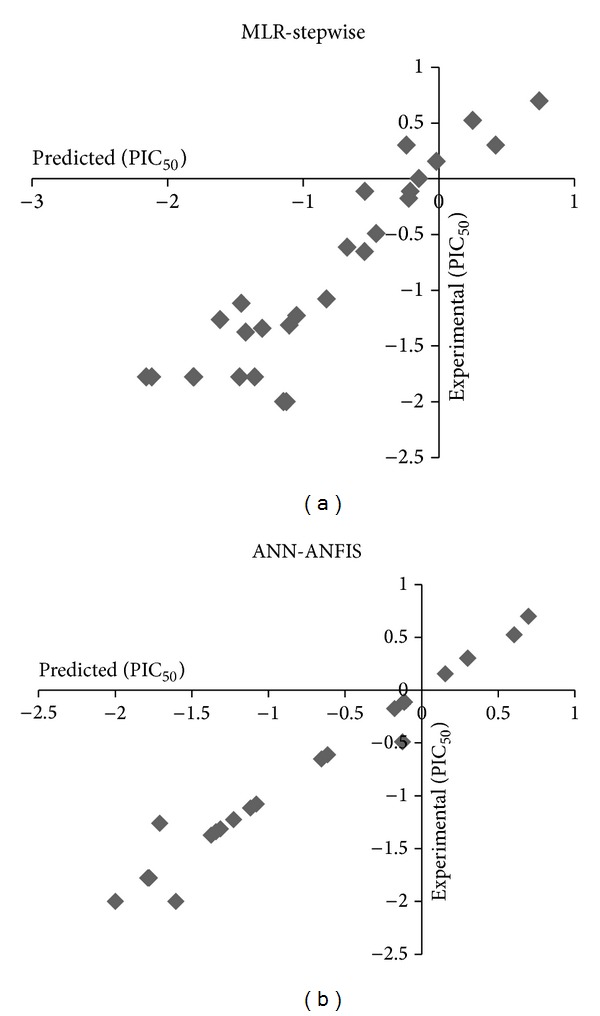
Results and comparison of MLR-stepwise and ANN-ANFIS models.

The underlying basis of this study has focused on elucidation of the molecular structure and anticancer activity of these compounds with two methods of MLR-stepwise and ANN-ANFIS. Here QSAR studies were confident to receptor-independent (RI) QSAR analyses and the geometry of the receptor is neglected [35]. The data set was divided into training and test sets. The test set was used for external validation. The ANN-ANFIS model with high statistical significance has better capability to predict anticancer activity of new compounds series of this family (Figure 3).

The QSAR models should be interpretable and it is important to explain the selected descriptors [58]. Definition of each selected descriptor was presented here. Anticancer activity of this series of compounds could not be attributed to one or two structural features of the molecules and the anticancer activity is the product of optimizing a collection of descriptors. It has been observed that in MLR-stepwise model 2D autocorrelation, 3D-MoRSE, GETAWAY, topological, and WHIM descriptors have more effect on anticancer activity than quantum chemical ones. The values of the mean effect (MF) were calculated according to (3) to indicate the relative importance of these descriptors. Consider

(3) $$\begin{array}{c} {\text{MF}}_{j}=\frac{\beta_{j}\sum_{i=1}^{i=n}d_{ij}}{\sum_{j}^{m}\beta_{j}\sum_{i}^{n}d_{ij}}, \end{array}$$

where MF_*j*_ represents the mean effect of the considered descriptor *j*, *b*_*j*_ is the coefficient of the descriptor *j*, *d*_*ij*_ stands for the value of the target descriptors for each molecule, and, eventually, *m* is the descriptors number in the model [46, 59].

MF values are 0.506, 0.415, 0.048, and 0.030, 0 for G3s, CIC1, H8m, Mor28u, and MATS8e, respectively. The high value of mean effect for G3s shows the significance of this descriptor in the model. G3s is one of the global WHIM descriptors which display a positive sign on PIC_50_. Weighted holistic invariant molecular (WHIM) descriptors are geometrical descriptors which show molecular 3D information regarding molecular size, shape, symmetry, and atom distribution. WHIM descriptors are suitable for complex properties. In G3s, WHIM weighted covariance matrixes were provided by the electrotopological state indexes of Kier and Hall [38]. CIC1 (complementary information content with neighborhood symmetry of 1-order) is the second order of importance. It is a topological descriptor. Topological indexes are single indexes derived from a molecular graph which can be sensitive to one or more structural features of the molecule such as size, shape, symmetry, branching, and cyclicity. This descriptor shows the molecular symmetry by measuring the neighborhood of the atoms (through the value of the vertex degrees) located at a first-order distance (one single bond) of a considered atom, for each vertex in G [38, 60]. MATS8e (Moreau autocorrelation— lag8/weighted by atomic Sanderson electronegativities) is one of the 2D-autocorrelation descriptors by Broto-Moreau calculated from the molecular graph by summing the products of atom weights of the terminal atoms of all the paths of length 8, using the Sanderson electronegativities as weighting scheme (the lag). Variation in toxicity as a function of position and nature of the substituent is determined by 2D parameters. It shows that replacing hydroxyl group with methoxy in different positions of the salen ligand plays a crucial role regarding toxicity. It is well known that the stereo chemical moieties of the investigated compounds could affect biological activity so 2D models of molecules can provide stereo chemical information [38, 61]. H8m (H autocorrelation of lag8/weighted by atomic masses) is of the GETAWAY descriptors which are geometrical descriptors which encode information on the effective position of substituents and fragments in the molecular space. In fact GETAWAY descriptors encode both the geometrical information given by the influence molecular matrix and the topological information given by the molecular graph [38, 62]. Mor28u (Signal 28/unweighted) is one of the 3D-MoRSE descriptors which represent structures based on electron diffraction descriptors so they can reveal the skeleton and substituents information for a molecule. Various physicochemical properties such as atomic mass, partial atomic charges, and atomic polarizability were considered to present high flexibility of a molecule. The form of the intensity distribution *I*(*s*) is given by

(4) $$\begin{array}{c} I\left(s\right)=\overset{N}{\underset{i=2}{\sum }}\overset{i-1}{\underset{j=1}{\sum }}A_{i}A_{j}\frac{\sin{sr}_{ij}}{{sr}_{ij}},\;s=0,\ldots ,31.0\;{Å}^{-1}, \end{array}$$

where *N* is the number of atoms, *r*_*ij*_ is the distance between atoms *i* and *j*, *A*_*i*_ can be any atomic property of atom *i* such as atomic number, mass, partial atomic charge, or atomic polarizability, and *s* is a reciprocal distance. The value of *s* was considered only at discrete positions within a certain range, between 0 and 31 Å^−1^. For Mor28u, an atomic mass weighted scheme was used and *s* was equal to 27 Å^−1^ [63, 64].

Two QSAR models were built here using various types of descriptors. In nonlinear model Mor28p, Signal28/weighted by atomic polarizabilities is one of the 3D-MORSE descriptors whose autocorrelation vectors are weighted by atom polarizabilities. SPI (superpendentic index) is one of the topological descriptors which derived from the *H*-depleted molecular graph and is calculated according to the following:

(5) $$\begin{array}{c} \int_{p}={\left(\overset{A}{\underset{i=1}{\sum }}\pi_{m}d_{im}\right)}^{1/2}, \end{array}$$

where *d* is the topological distances, that is, row of the pendent matrix, and *m* is the number of terminal vertices, that is, the column of the pendent matrix [65, 66]. RDF110m is one of the 3D-radial distribution function (RDF) descriptors which were proposed based on a radial distribution function. The radial distribution function is probability distribution to find an atom in a spherical volume of radius *R*. RDF descriptors are independent of the size and rotation of the entire molecule. They describe the steric hindrance or the structure/activity properties of a molecule. The general equation of the radial distribution function is in accordance with the following:

(6) $$\begin{array}{c} g\left(R\right)=f\cdot \overset{A-1}{\underset{i=1}{\sum }}\overset{A}{\underset{j=i+1}{\sum }}w_{i}\cdot w_{j}\cdot e^{-\beta \cdot {\left(R-r_{ij}\right)}^{2}}, \end{array}$$

where *f* is a scaling factor, *w* is the characteristic atomic properties of the atoms *i* and *j*, *r*_*ij*_ is the interatomic distance between the *i*th and *j*th atom, and *A* is the number of atoms. The exponential term contains the distance *r*_*ij*_ between the atoms *i* and *j* and the smoothing parameter *β* that defines the probability distribution of the individual interatomic distances. *β* can be interpreted as a temperature factor which defines the movement of atoms. The RDF descriptor provides valuable information about the bond distances, ring types, planar and nonplanar systems, and atom types [38, 59]. SPCN8 is shielding NMR (ppm) of the nitrogen8 which is calculated by Gaussian 03. Final MATS5v (Moran autocorrelation—lag 5/weighted by atomic Sanderson electronegativities) is one of the 2D-autocorrelation descriptors.

**Figure 4:**
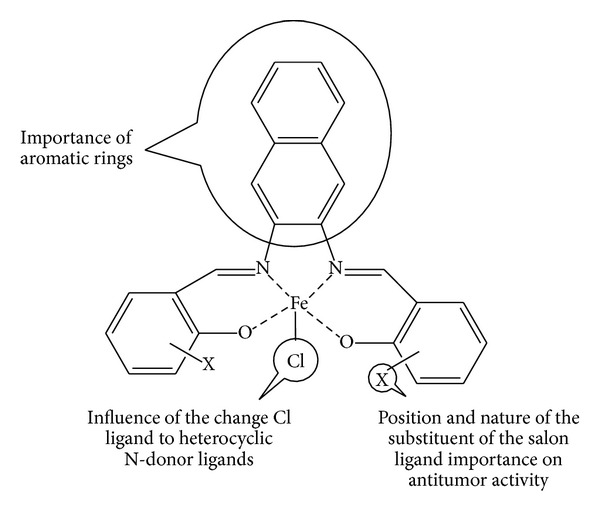
Importance of the position and nature of the substituent of the salen ligand.

This study provides deeper insight into the antitumor activity of the Fe(III)-salen-like complexes. Based on the above discussion the anticancer activity of this compound is mainly dependent on the geometrical parameters and position and nature of the substituent of the salen ligand. Data analysis shows that the increase in aromatic rings on the bridge between D-amino groups causes more activity of the complex. Geometrical parameters are important in the ligand transportation through the cell membrane. Also change in the position of the substituent of the salen ligand changes the anticancer activity. The nature of the substituent has a sharp effect on the biological activity. Our studies on the influence of Cl ligand replacing the heterocyclic N-donor ligands show that 1H-tetrazol-5-amin(Hatz) increases in activity. Results show that change of Cl ligand on the heterocyclic N-donor ligands has a minor effect compared to aromatic group replacement on the anticancer activity which is shown in Figure 4.

## 4. Conclusion

Some of iron(III)-salen complexes have a very desirable anticancer activity against MCF7 cells. Their anticancer activity is the result of optimizing a collection of descriptors, considering that acquired results could not attribute the anticancer activity to one or two special structural features. Also, the results of this study show the high ability of nonlinear methods which resulted from fuzzy logic and neural network in anticipating the anticancer activity of new series of salen complexes such as iron(III). The ANN-ANFIS model with high statistical significance has better capability to predict anticancer activity of the new compounds series of this family. The results show the importance of the geometrical parameters and position and nature of the substituent of the salen ligand on the anticancer activity.

## Figures and Tables

**Table 1:** Experimental and predicted values of PIC_50_ for various salen complexes by MLR and ANN models.

Structure	PIC_50_ experimental	PIC_50 _ predicted	
		ANN	MLR
1	−1.34227	−1.30399	−1.34220
2	−1.77815	−1.81275	−1.77810
3	−1.77815	−2.15957	−1.77810
4	−1.77815	−2.11785	−1.77810
5	−1.77815	−1.47180	−1.77820
6	−1.07918	−0.82961	−1.07920
7	−0.65321	−0.54961	−0.65320
8	−0.11394	−0.54747	−0.11390
9	0.52288	0.24813	0.60580
10	−1.77815	−1.80770	−1.78810
11	−1.77815	−1.35897	−1.78810
12	−0.61278	−0.67836	−0.61280
13	0.1549	−0.01870	0.15500
14	−0.49136	−0.46266	−0.12430
15	0.30103	−0.24032	0.30110
16	0.69897	0.73866	0.69890
17	−2.00000	−1.12536	−1.60450
18	−2.00000	−1.14808	−2.00000
19	−0.17609	−0.22396	−0.17610
20	0.30103	0.41782	0.30100
21	−0.11394	−0.21354	−0.11390
22	−1.37475	−1.42517	−1.37470
23	−1.11727	−1.45753	−1.11730
24	−1.31387	−1.10417	−1.31390
25	−1.26245	−1.61461	−1.71060
26	−1.22789	−1.04990	−1.22790
Cisplatin	−1.25527		

PIC_50_ = −log⁡(IC_50_).

**Table 2:** Descriptors used in MLR.

Number	Symbol	Chemical meaning	Type
1	MATS8e	Moreau autocorrelation—lag8/weighted by atomic sanderson electronegativities	2D autocorrelation
2	Mor28u	Signal 28/unweighted	3D-MoRSE
3	H8m	H autocorrelation of lag8/weighted by atomic masses	GETAWAY
4	CIC1	Complementary information content (neighborhood symmetry of 1-order)	Topological
5	G3s	3st component symmetry directional WHIM index/ weighted by atomic electrotopological states	WHIM

**Table 3:** Correlation coefficient matrix of the selected descriptors.

	MATS8e	Mor28u	H8m	CIC1	G3s
MATS8e	1	−0.273	0.270	0.026	−0.283
Mor28u		1	−0.109	−0.668	0.347
H8m			1	−0.124	−0.259
CIC1				1	−0.009
G3s					1

**Table 4:** Five most efficient descriptors selected by ANFIS models.

No.	Symbol	Chemical meaning	Type
1	Mor28p	Signal28/weighted by atomic polarizabilities	3D-MoRSE
2	SPI	Superpendentic index	Topological
3	RDF110m	Radial distribution function 11.0/weighted by atomic masses	RDF
4	SPCN8	Shielding NMR (ppm) of Nitrogen8	NMR
5	MATS5v	Moreau autocorrelation—lag5/weighted by atomic van der Waals volumes	2D autocorrelation

**Table 5:** Results and validation of QSAR models.

	*R*_train_^2^	*Q* ^2^_LOO_	RMSE
MLR-stepwise	0.863	0.769	0.342
ANN-ANFIS	0.999	0.820	0.138
